# Supplementary material for: A control oriented strategy of disruption prediction to avoid the configuration collapse of tokamak reactors
Source: Nat Commun. 2024 Mar 18;15:2424. doi: 10.1038/s41467-024-46242-7 (PMC10948913; doi:10.1038/s41467-024-46242-7)
Supplement: Supplementary file 1 — Supplementary Information [file 41467_2024_46242_MOESM1_ESM.pdf]

SUPPLEMENTARY MATERIAL TO

A Control Oriented Strategy of Disruption Prediction to Avoid the Configuration Collapse of Tokamak Reactors

Andrea Murari[1,2*], Riccardo Rossi[3*], Teddy Craciunescu[4], Jesús Vega[5], JET Contributors[£] and Michela Gelfusa[3]

EUROfusion Consortium, JET, Culham Science Centre, Abingdon, OX14 3DB, UK

[1] *Consorzio RFX (CNR, ENEA, INFN, Università di Padova, Acciaierie Venete SpA), Corso Stati Uniti 4, 35127 Padova, Italy.*
[2] *Istituto per la Scienza e la Tecnologia dei Plasmi, CNR, Padova, Italy*
[3] *Department of Industrial Engineering, University of Rome "Tor Vergata", via del Politecnico 1, Roma, Italy*
[4] *National Institute for Laser, Plasma and Radiation Physics, Magurele-Bucharest, Romania*
[5] *Laboratorio Nacional de Fusión, CIEMAT. Av. Complutense 40. 28040 Madrid. Spain*

**These authors contributed equally to the paper*

£ A list of authors and their affiliations appears at the end of the paper.

Corresponding author: gelfusa@ing.uniroma2.it

Supplementary Figures

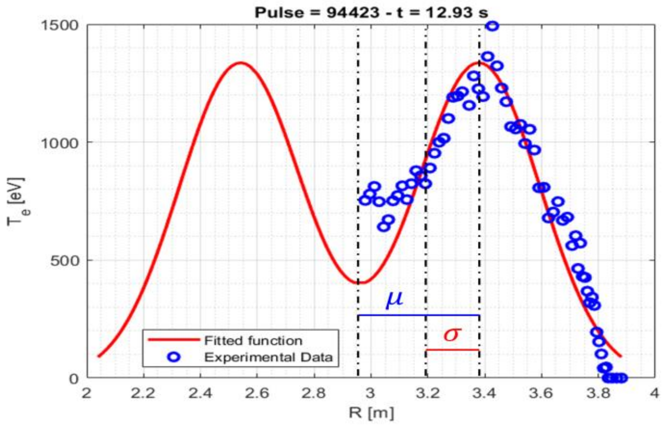

Supplementary Figure 1 | Hollow profile. An example of hollow temperature profile, the Gaussian fit and the quantities entering in the proposed GFH hollowness indicator.

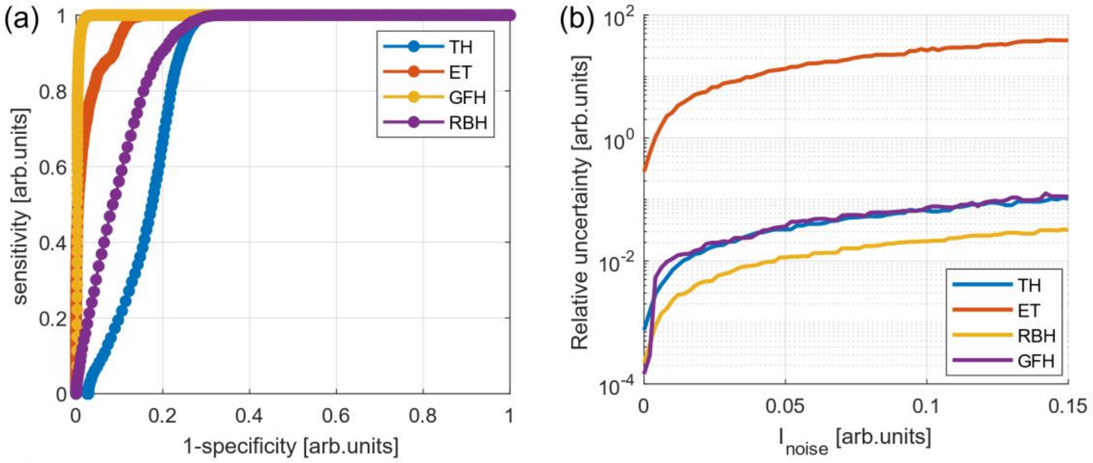

(c)

Indicators	TH	ET	RBH	GFH
**Classification Performances**	Sensitivity: ~93%<br>Specificity: ~75%<br>Accuracy: ~80%	Sensitivity: ~85%<br>Specificity: ~95%<br>Accuracy: ~92%	Sensitivity: ~82%<br>Specificity: ~84%<br>Accuracy: ~83%	Sensitivity: ~98%<br>Specificity: ~99%<br>Accuracy: ~98%
**Regression Performances (Expected vs measured)**	Linear R^2 ~ 52%	Linear R^2 ~ 47%	Linear R^2 ~ 58%	Linear R^2 ~ 96%
**Sensitivity to noise**	Low	High	Very low	Low
**Sensitivity to outliers**	Max perturbation around 5%	High perturbation for specific channels	Max perturbation around 1%	Max perturbation around 5%
**Computational time**	~14 µs	~3 µs	~16 µs	~1.8 ms<br>(can be decreased)

Supplementary Figure 2 | Hollowness indicator statistics. a. the ROC (Receiver Operating Characteristics) curve comparing the overall performances of the investigated indicators. **b.** the sensitivity of the indicators to the amplitude of Gaussian noise, whose standard deviation is reported on the x axis. The ordinate axis shows the relative uncertainty, calculated as the standard deviation divided by the average value. **c.** Comparison of the GFH performance and the other indicators (TH, ET, RBH) reported in the literature [1].

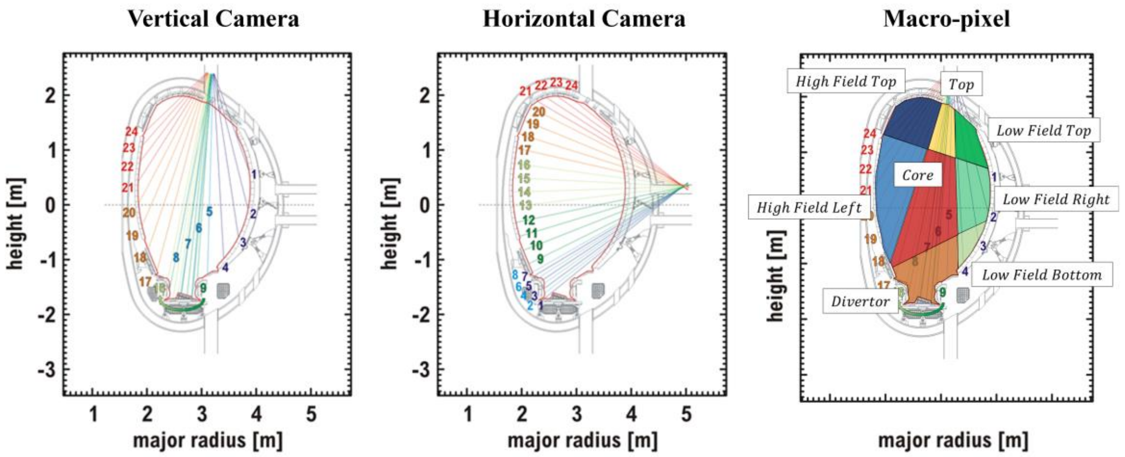

Supplementary Figure 3 | Fast time resolution bolometry. Left hand and central images: layout of JET bolometry. Different colours indicate the lines of sight use to form the three macro-views for each camera. Right hand image: the 8 macro-pixels resulting from intersecting the macro-views.

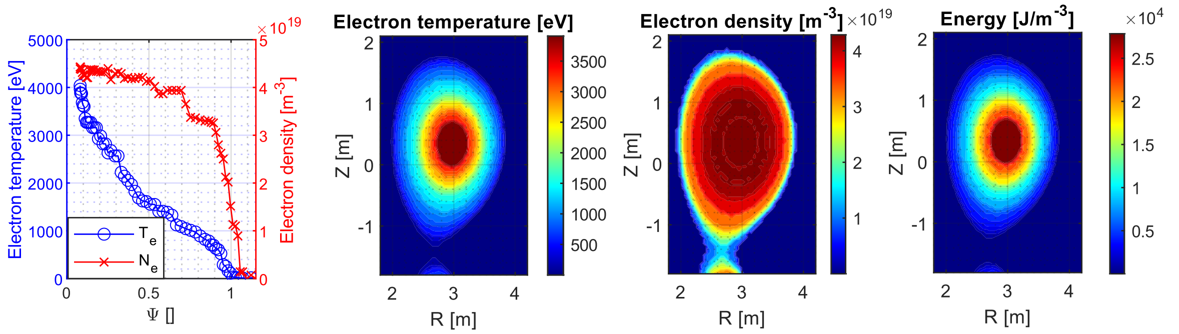

Supplementary Figure 4 | Reconstruction of the plasma fields. Reconstruction of the temperature, density and pressure fields for discharge number 95998 at t = 18 s.

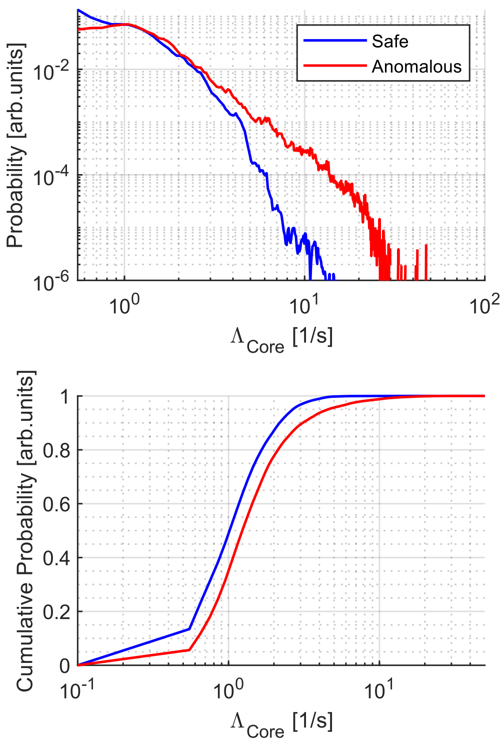

Supplementary Figure 5 | Radiation anomaly detection. Example of probability distribution of Λ_{core}. Top: pdf. Bottom: CDF.

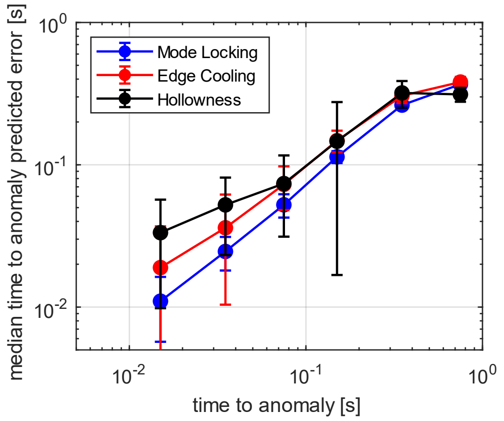

Supplementary Figure 6 | Time to anomaly prediction error. The median error in the estimate time to anomaly vs the interval elapsing before the actual onset of the anomaly. In the case of the locked mode the actual time of the anomaly onset is considered the beginning of the current quench. Errors bars are calculated as standard deviations.

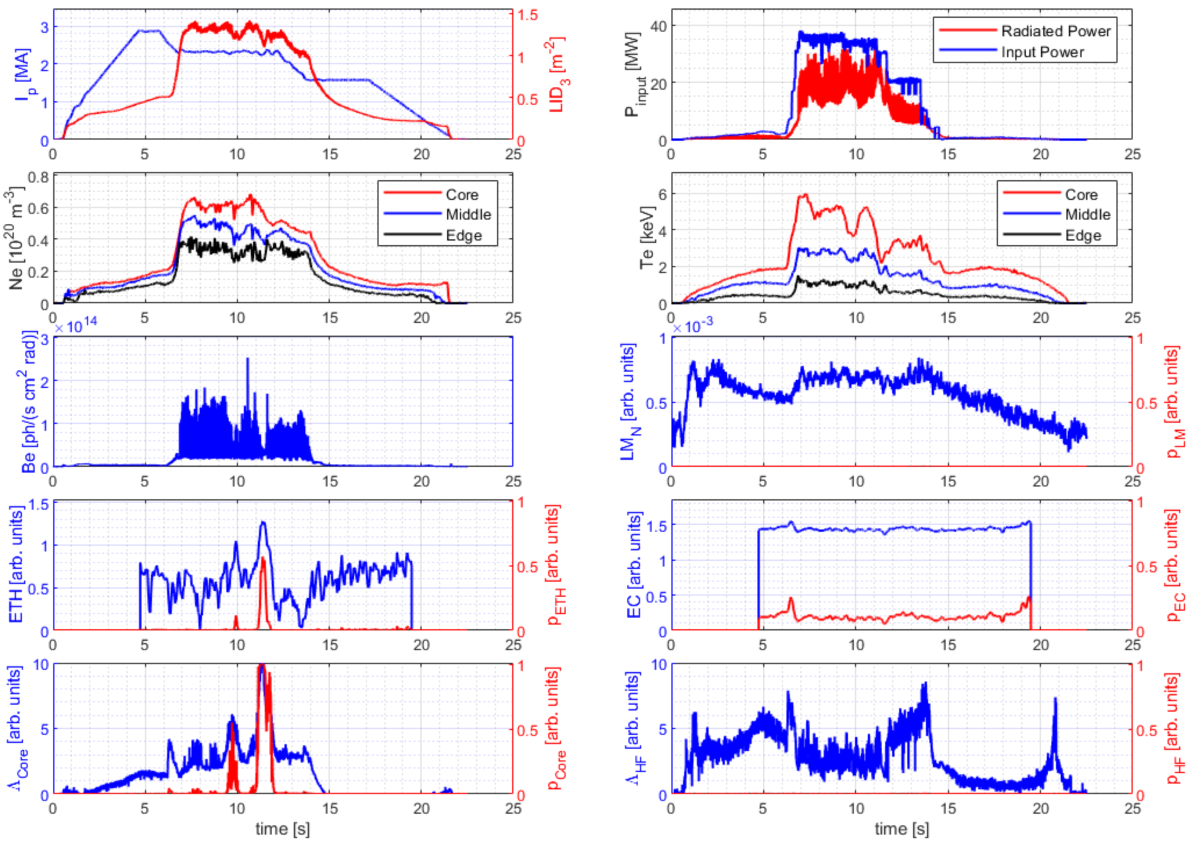

Supplementary Figure 7 | Pulse 96527. Evolution of the main plasma parameters for discharge #96527. The first hollow profile is detected at t ~ 11.05 s, the most pronounced hollowness intensity at t ~ 11.30 s.

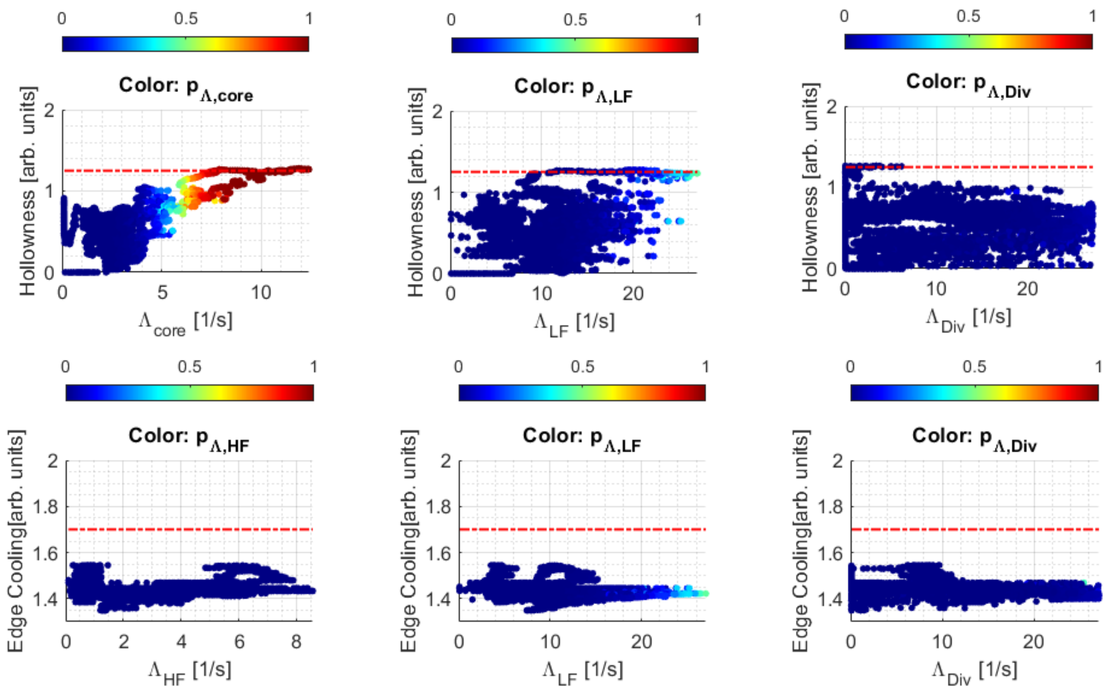

Supplementary Figure 8 | Temperature and Radiation paths. Probabilistic maps of edge cooling and temperature hollowness vs the λ indicators of the various regions for discharge 96527, showing that indeed the cause of the anomalous temperature profile is excessive radiation in the core.

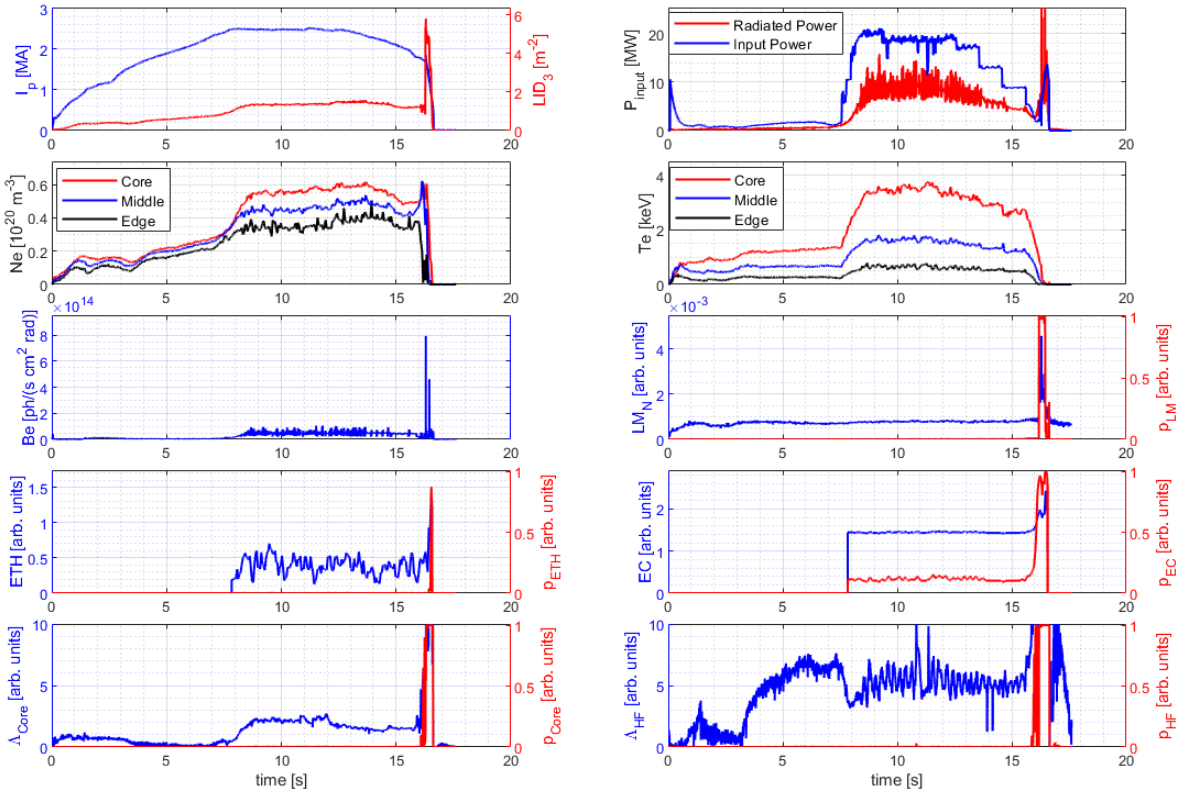

Supplementary Figure 9 | Pulse 95993. Evolution of the main plasma parameters for discharge #95993.

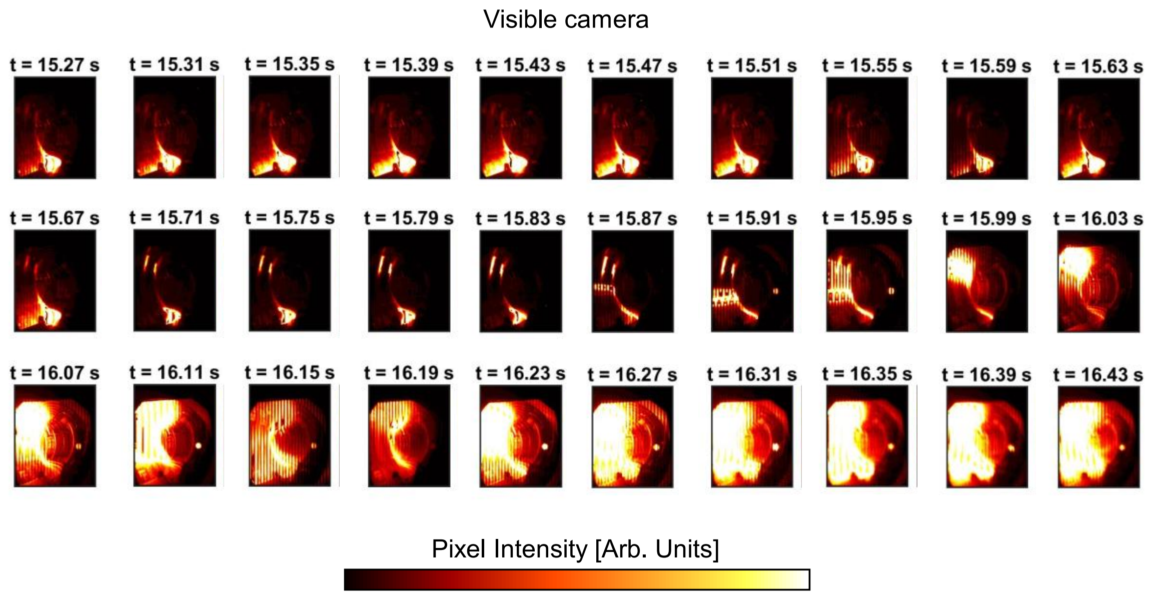

Supplementary Figure 10 | MARFE. MARFE seen by the frames of a visible camera in discharge #95993.

Supplementary Tables

Supplementary Table 1| Edge Cooling indicator statistics. Comparison of the edge cooling indicator CBC and the main alternatives reported in the literature. The indicators reported in the literature (EC, RBEC and EC) are described in detail in [1]. The CBC indicator outperforms all the others according to all the metrics, except the computational time, which however is perfectly adequate to perform even the most stringent real time actions.

**Indicators**	**EC (ρ_{cool} < 0.35)**	**RBEC**	**CBC**	**EDC**
**Classification Performances**	Sensitivity: ~82% (~93%) Specificity: ~90% (~93%) Accuracy: ~84% (~93%)	Sensitivity: ~92% Specificity: ~84% Accuracy: ~92%	Sensitivity: ~93% Specificity: ~93% Accuracy: ~93%	Sensitivity: ~91% Specificity: ~79% Accuracy: ~86%
**Regression Performances (Expected vs measured)**	Linear R^2 ~ 38% (~ 4%)	Linear R^2 ~ 78%	Linear R^2 ~ 94%	Linear R^2 ~ 78%
**Sensitivity to noise**	High (low)	Very low	Low	Middle
**Sensitivity to outliers**	Max perturbation around 5%	Max perturbation around 1%	Max perturbation around 2%	Max perturbation around 5%
**Computational time**	~14 µs	~16 µs	~100 µs	~45 µs

Supplementary Table 2 | Error of estimated energy. Average difference between the estimate of the macro-pixels' energy calculated with equation (12) and by fitting the measurements of the High-Resolution Thomson Scattering (HRTS) with a second order polynomial.

	Relative Error using HRTS
**LFB**	17.03%
**LFR**	8.47%
**LFT**	23.00%
**Core**	4.08%
**Div**	13.85%
**Top**	17.41%
**HFL**	11.80%
**HFT**	23.38%

Supplementary Table 3 | JET alarms vs New Predictor. Comparison of JET alarms and the ones obtained with the tools developed and described in the present work. The table compares JET control system actions with the ones that would have been possible with the proposed tools.

	Predictor Actions
**Disruptive JET DMV**	Mitigated:3
	Prevented:5
	Avoided: 28
	Total: 36
**Disruptive JET JTT**	Mitigated:1
	Prevented:0
	Avoided: 18
	Total: 19
**Disruptive JET no actions**	Mitigated:5
	Prevented:4
	Avoided: 11
	Total: 20
**Safe JET JTT**	Mitigated: 7
	Prevented: 0
	Avoided: 10
	Total: 33
**Safe JET no actions**	Mitigated:0
	Prevented:0
	Avoided: 0
	Total: 178

Supplementary Table 4 | JET DMV vs New Predictor. Comparison of JET alarms and the ones obtained with the tools developed and described in the present work. The table reports the anticipation times of the developed predictors compared to the firing of the Massive Gas Injection valve (DMV).

	Disruptive Time vs DMV
**Mean [ms]**	Mitigated: 481
	Prevented: 135
	Avoided: 1115
**Median [ms]**	Mitigated: 165
	Prevented: 118
	Avoided: 689
**Min [ms]**	Mitigated: 25
	Prevented: 80
	Avoided: 135

References

[1] Rossi R.et al "*Development of robust indicators for the identification of electron temperature profile anomalies and application to JET*" Plasma Physics and Controlled Fusion, Volume 64, Issue 4, id.045002, 10.1088/1361-6587/ac4d3b
